# Halolysin SptA, a Serine Protease, Contributes to Growth-Phase Transition of Haloarchaeon *Natrinema* sp. J7-2, and Its Expression Involves Cooperative Action of Multiple *Cis*-Regulatory Elements

**DOI:** 10.3389/fmicb.2018.01799

**Published:** 2018-08-03

**Authors:** Moran Li, Jing Yin, Sha Mei, Xuhong Wang, Xiao-Feng Tang, Bing Tang

**Affiliations:** ^1^State Key Laboratory of Virology, Hubei Key Laboratory of Cell Homeostasis, College of Life Sciences, Wuhan University, Wuhan, China; ^2^Hubei Provincial Cooperative Innovation Center of Industrial Fermentation, Wuhan, China

**Keywords:** haloarchaea, serine protease, growth-phase transition, *cis*-acting element, promoter

## Abstract

Many haloarchaea produce extracellular subtilisin-like proteases (halolysins) during late log phase; however, the physiological function and regulatory mechanism of growth phase-dependent production of halolysins are unknown. Halolysin SptA, the major extracellular protease of *Natrinema* sp. J7-2, is capable of intracellular self-activation to affect haloarchaeal growth. Here, we report that deletion of *sptA* leads to loss of extracellular and intracellular protease activities against azocasein and/or suc-AAPF-pNA, as well as a change in growth-phase transition of the haloarchaeon. Our results suggest that SptA is important for strain J7-2 to enter the stationary and death phases. Deletion and mutational analyses of the 5′-flanking region of *sptA* revealed two partially overlapping, semi-palindromic sequences upstream of the TATA box act as positive and negative *cis*-regulatory elements, respectively, to mediate *sptA* expression in late log phase. Additionally, a negative *cis*-regulatory element covering WW motif and a distant enhancer contribute to the modulation of *sptA* expression. Our results demonstrate that SptA functions both extracellularly and intracellularly, and that *sptA* expression relies on the cooperative action of multiple *cis*-regulatory elements, allowing SptA to exert its function properly at different growth stages in strain J7-2.

## Introduction

Haloarchaea generally require 2–5.2 M NaCl for growth and thrive in hypersaline environments such as solar salterns, salt lakes, and salt deposits (Grant, 2004). Many haloarchaea produce extracellular proteases, most of which are closely related to the subtilisin-like serine protease (subtilase) superfamily, known as halolysins (Siezen and Leunissen, 1997; De Castro et al., 2006). Several halolysins have been characterized, including 172P1 from *Natrialba asiatica* (Kamekura et al., 1992), R4 from *Haloferax mediterranei* (Kamekura et al., 1996), SptA and SptC from *Natrinema* sp. J7 (Shi et al., 2006; Zhang et al., 2014), and Nep from *Natrialba magadii* (De Castro et al., 2008). In addition, an increasing number of halolysin-encoding genes have been identified in sequenced haloarchaeal genomes, highlighting the importance of halolysins in the haloarchaea. Halolysins are synthesized as inactive precursors composed of a signal peptide, an N-terminal propeptide, a subtilisin-like catalytic domain, and a C-terminal extension (CTE). Precursors of halolysins are translocated across the cytoplasmic membrane via the twin-arginine translocation (Tat) pathway, which is used for the secretion of folded proteins (Bolhuis, 2002; Rose et al., 2002; Shi et al., 2006). After the removal of the signal peptide by a signal peptidase, the resulting proform undergoes autocleavage of its N-terminal propeptide to generate the active mature halolysin containing the catalytic domain and the CTE (Ruiz et al., 2012; Du et al., 2015). Notably, it was shown that in their native hosts, halolysins (e.g., 172P1, Nep, and SptA) are produced during late log phase and reach peak production when the culture enters stationary phase (Kamekura and Seno, 1990; Paggi et al., 2010; Du et al., 2015). Many halolysin-like serine protease genes have been annotated in the genomes of *Halobacterium salinarium* and *Natronococcus occultus*, and the extracellular serine proteases of the two strains are also produced during late log phase (Norberg and von Hofsten, 1969; Elsztein et al., 2001). Additionally, the production of Nep in *Nab. magadii* is repressed by ammonium (D’Alessandro et al., 2007). These findings show that the expression levels of halolysins and extracellular serine proteases in haloarchaea are strictly regulated. It has been suggested that the expression of *nep* is upregulated in response to regulatory factors (metabolites and/or regulatory molecules) present in high-density cultures of *Nab. magadii* (Paggi et al., 2010), and that the production of the extracellular serine protease in *Ncc. occultus* is induced in a quorum sensing-dependent manner by autoinducer molecules (Paggi et al., 2003). Neither the putative regulatory factors for Nep expression nor the autoinducer molecules for the *Ncc. occultus* serine protease have been identified, however. The *cis*-acting and *trans*-acting factors in the regulation of growth phase-dependent halolysin production are still unclear. From a physiological viewpoint, the extracellular proteases of haloarchaea may serve a nutritional purpose by degrading external protein substrates to generate oligopeptide, dipeptide, and amino acid intermediates, which feed into the central metabolism (De Castro et al., 2006). However, the fact that halolysin production occurs during late log phase suggests that halolysins are not essential for the exponential growth of haloarchaea. The physiological roles of halolysins in the growth and survival of haloarchaea remain to be elucidated.

The haloarchaeon *Natrinema* sp. J7 was isolated from a salt mine in Hubei province, China (Shen and Chen, 1994) and grows optimally in the presence of 3.1–3.8 M NaCl. The complete genome of *Natrinema* sp. J7-2 [a subculture of strain J7 lacking the plasmid pHH205 (Ye et al., 2003)] has been sequenced (Feng et al., 2012). Halolysin SptA, the major extracellular protease of *Natrinema* sp. J7-2, is produced and secreted primarily during late log phase (Feng et al., 2014; Du et al., 2015). The enzymatic properties, autocatalytic activation, and Tat-dependent secretion mechanisms of SptA have been characterized (Shi et al., 2006; Xu et al., 2011; Du et al., 2015). Recently, the core promoter and transcript of the *sptA* gene were experimentally determined (Tang et al., 2016). The results showed that the *sptA* core promoter contains some highly conserved, *cis*-acting elements of haloarchaeal promoters, including a transcription factor B (TFB) recognition element (BRE) motif, a TATA box recognized by the TATA-binding protein (TBP), and a WW motif that possibly interacts with TFB and RNA polymerase (RNAP) (Brenneis et al., 2007). Notably, although *Natrinema* sp. J7-2 could efficiently secrete SptA via the Tat pathway, it accumulated active mature SptA intracellularly during the stationary and death phases. When SptA was expressed in *Hfx. volcanii*, mutation of the Tat signal peptide retarded the secretion of recombinant SptA, leading to intracellular accumulation of the active mature enzyme and subsequent cell death (Du et al., 2015). These findings suggest that the intracellular activation of a Tat-dependent halolysin affects haloarchaeal growth.

In this study, *sptA*-deletion mutants of *Natrinema* sp. J7-2 were constructed and they were phenotypically characterized in order to clarify the physiological functions of SptA. The results showed that halolysin SptA influences the growth characteristics of *Natrinema* sp. J7-2. Furthermore, multiple *cis*-regulatory elements involved in the regulation of the growth phase-dependent production of SptA were identified by deletion and site-directed mutagenesis. We discuss the mechanism by which the growth phase-dependent synthesis of SptA is regulated based on our results.

## Materials and Methods

### Strains and Growth Conditions

The bacterial and haloarchaeal strains used in this study are listed in Supplementary Table S1. *Natrinema* sp. J7-2, a subculture of *Natrinema* sp. J7 (CCTCC AB91141) lacking the plasmid pHH205 (Ye et al., 2003; Feng et al., 2012), and its derivatives were grown aerobically at 37°C with shaking at 180 rpm in liquid modified growth medium with 23% total salts (23% MGM) containing 1 g yeast extract, 5 g peptone, 184 g NaCl, 23 g MgCl_2_.6H_2_O, 26.8 g MgSO_4_.7H_2_O, 5.4 g KCl, 0.42 g CaCl_2_, and 3.8 ml 1 M Tris-HCl (pH 7.5) per liter^1^. When necessary, 5 μg ml^-1^ mevinolin was added into the culture medium to cultivate haloarchaeal strains harboring mevinolin-resistant plasmids. To determine the number of viable cells, the haloarchaeal cultures were plated on 18% MGM (1 g yeast extract, 5 g peptone, 144 g NaCl, 18 g MgCl_2_.6H_2_O, 21 g MgSO_4_.7H_2_O, 4.2 g KCl, 0.33 g CaCl_2_, and 3 ml 1 M Tris-HCl [pH 7.5] per liter) agar plates and incubated at 37°C for 5–10 days. *Escherichia coli* DH5α and *E. coli* JM110 were used as hosts for plasmid construction and were grown at 37°C in Luria-Bertani medium supplemented with ampicillin (100 μg ml^-1^) as needed.

### Plasmid Construction and Mutagenesis

The plasmids and primers used in this study are listed in Supplementary Tables S1, S2, respectively. The genomic DNA of *Natrinema* sp. J7-2 or its mutants was prepared according to the method of Kamekura et al. (1992). For construction of the knockout plasmid pNBK-*Aud* for the core promoter and ORF region of *sptA*, the 349 bp region upstream of the *sptA* core promoter and the 376 bp region immediately downstream of the *sptA* ORF were amplified from *Natrinema* sp. J7-2 genomic DNA by PCR with primer pairs SptAus-F/SptAus-R and SptAds-F/SptAds-R, respectively. The two PCR fragments were fused by the overlapping extension PCR method (Bian et al., 2006) with primer pair SptAus-F/SptAds-R and then cloned into the BglII-HindIII restriction site of the vector pNBK07 (Wang et al., 2016). The knockout plasmid pNBK-3′UTR*ud* for the 3′UTR region of *sptA* was constructed in the same way as pNBK-*Aud*, except that the genomic DNA of the mutant Δ*sptA1* (see below) was used as the template, and the primer pairs SptAus-F/3′UTRus-R and 3′UTRds-F/3′UTRds-R were used to amplify the 3′UTR flanking regions. To construct the complementary vector pSHS-*sptA*, the core promoter and ORF region of *sptA* was amplified from *Natrinema* sp. J7-2 genomic DNA by PCR with primer pair SptA-BamHI-F/SptA-HindIII-R and then cloned into the BamHI-HindIII site of the vector pYC-SHSmcs (Supplementary Figure S1) (Wang, 2016).

For truncation analysis of the 5′-flanking region of *sptA*, DNA fragments from different upstream positions (-335, -215, -174, -142, -116, -82, -63, -51, and -43) to the 3′-end (+1925) of the *sptA* transcript region were amplified from *Natrinema* sp. J7-2 genomic DNA by PCR using different forward primers (up335-F to up43-F) in combination with the common reverse primer 3′UTR-HindIII-R. The PCR products were individually cloned into the BamHI-HindIII restriction site of pYC-SHSmcs, yielding the plasmids pD335 to pD43 with *sptA* as a reporter gene. When *bgaH* was used as a reporter gene to conduct the truncation analysis, the megaprimer PCR method (Sarkar and Sommer, 1990) was employed to replace *sptA* with *bgaH.* Briefly, *Natrinema* sp. J7-2 genomic DNA template was combined with different forward primers (up215-F to up43-F) and a common reverse primer (up-bgaH-R) to amplify *sptA* 5′-flanking region of different lengths (megaprimers). Subsequently, *bgaH* was amplified from plasmid pST1 (Tang et al., 2016) using the megaprimer and the primer bgaH-HindIII-R. The PCR products were individually cloned into the BamHI-HindIII restriction site of pYC-SHSmcs to generate the plasmids pD215b to pD43b. The megaprimer PCR method was also used to introduce site-directed point mutations into the 5′-flanking region of *sptA*. Using plasmid pD215 as the template, megaprimers were prepared by PCR with a common forward primer (up215-F) and different reverse primers (m1-R to m16-R) containing the target mutations. The full-length sequences were amplified from pD215 with the megaprimers and the reverse primer 3′UTR-HindIII-R and then inserted into the BamHI-HindIII restriction site of pYC-SHSmcs to generate the plasmids pM1 to pM16. A second round of megaprimer PCR was conducted to obtain plasmids with combined mutations (e.g., pM3-6, pM3-6-11). The sequences of all recombinant plasmids were verified by DNA sequencing.

### Construction of *sptA*-Deletion Mutants

The deletion of *sptA* in *Natrinema* sp. J7-2 was carried out using the pop-in/pop-out method (Liu et al., 2011). Briefly, the knockout plasmid pNBK-*Aud* was amplified in *E. coli* DH5α and then transferred into *E. coil* JM110 to prepare non-methylated plasmid. Thereafter, the non-methylated pNBK-*Aud* was transferred into *Natrinema* sp. J7-2 as described previously (Lv et al., 2015). A single homologous recombination event between one of the flanking regions on pNBK-*Aud* and the chromosome (pop-in) was selected by growing the transformants on 18% MGM agar plates with 5 μg ml^-1^ mevinolin. The selected transformants were successively subcultured ∼ 10 times in 23% MGM to allow for a second homologous recombination event leading to the excision of the plasmid backbone from the chromosome (pop-out), thereby yielding the mutant strain Δ*sptA1* with a deletion of the core promoter and ORF region of *sptA*. Using the same method, the 3′UTR region of *sptA* in the chromosome of the Δ*sptA1* mutant was further deleted with the knockout plasmid pNBK-3′UTR*ud* to construct the mutant Δ*sptA2* that had a deletion of the core promoter and transcript region of *sptA*. The mutants Δ*sptA1* and Δ*sptA2* were confirmed by PCR analysis with the internal primer pairs SptA-F/SptA-R and 3′UTR-F/3′UTR-R and the external primer pairs SptAus-F/SptAds-R and SptAus-F/3′UTRds-R followed by DNA sequencing.

### RNA Extraction and Quantitative Real-Time PCR (qRT-PCR) Analysis

Recombinant plasmids extracted from *E. coli* JM110 were transferred into Δ*sptA2* cells, and the transformants were grown in 23% MGM until the mid-log (optical density at 600 nm [OD_600_] ∼ 0.6) and/or stationary phase (OD_600_ ∼ 1.0). Total RNA was isolated from the cells using RNAiso plus (Takara) according to the manufacturer’s protocol. Genomic DNA removal and reverse transcription were conducted using a PrimeScript RT reagent kit with gDNA Eraser (Perfect Real Time; Takara) according to the manufacturer’s instructions. The qRT-PCR was carried out in a 20 μl reaction mixture as described previously (Tang et al., 2016). The *sptA* transcript levels were determined using the primer pair SptAQRT-F/SptAQRT-R. As an internal control, the 16S rRNA transcript levels were determined with the primer pair 16SrRNAQRT-F/16SrRNAQRT-R. The qRT-PCR results were calculated using the threshold cycle (2^-ΔΔC_T_^) method (Livak and Schmittgen, 2001). For quantification of the *sptA* transcript levels, 16S rRNA transcript *C_T_* values were used to normalize the *C_T_* values of the *sptA* transcripts.

### Proteolytic Activity Assay

The proteolytic activities of culture supernatants of *Natrinema* sp. J7-2 and its derivatives were assayed using azocasein (Sigma, St. Louis, MO, United States) as the substrate. Azocaseinolytic activity was determined at 37°C for 60 min in 200 μl reaction mixture containing 0.25% (w/v) azocasein and 100 μl culture supernatant in buffer A (50 mM Tris-HCl, 10 mM CaCl_2_, 3 M NaCl, pH 8.0). The reaction was terminated by adding 200 μl 40% (w/v) trichloroacetic acid (TCA) into the reaction mixture. After incubation at room temperature for 15 min, the sample was centrifuged at 13,400 ×*g* for 10 min, and the absorbance of the supernatant at 335 nm (*A_335_*) was measured in a 1 cm cell. One unit (U) of azocaseinolytic activity was defined as the amount of enzyme required to increase the *A_335_* by 0.01 per min under the assay conditions used.

The synthetic substrate *N*-succinyl-Ala-Ala-Pro-Phe-*p*-nitroanilide (suc-AAPF-pNA) (Sigma) was used to determine intracellular proteolytic activity. The cells of *Natrinema* sp. J7-2 and its derivatives were washed twice with buffer A and sonicated on ice in the same buffer. Subsequently, the cell extract was separated from the cell debris by centrifugation at 13,400 ×*g* for 10 min at 4°C and used to determine the proteolytic activity on 0.5 mM suc-AAPF-pNA at 37°C as described previously (Du et al., 2015).

### β-Galactosidase Activity Assay

The β-galactosidase activity of the strains harboring recombinant plasmids with the *bgaH* gene was determined using *o*-nitrophenyl-*β*-D-galactopyranoside (ONPG) (Sigma) as the substrate according to the method of Holmes et al. (1997). Briefly, the cells were washed three times with buffer B (50 mM Tris-HCl, 3 M NaCl, 10 μM MnCl_2_, 0.1% β-mercaptoethanol, pH 7.2) and then sonicated on ice in the same buffer. The cell extract was separated from the cell debris by centrifugation at 13,400 ×*g* for 10 min at 4°C and used for β-galactosidase activity assay at 37°C in buffer B containing 1.33 mM ONPG. The initial velocity of ONPG hydrolysis was monitored at 405 nm using a thermostat-controlled spectrophotometer (Cintra 10e, GBC, Australia), and the activity was calculated based on the extinction coefficient for *o*-nitrophenol (3,300 M^-1^ cm^-1^ at 405 nm). One unit (U) of β-galactosidase activity was defined as the amount of enzyme needed to produce 1 μM ρ-nitrophenol per min under the assay conditions described.

### SDS-PAGE and Immunoblot Analysis

SDS-PAGE was carried out according to the method of King and Laemmli (1971). To prevent self-degradation of the protease during sample preparation, proteins were precipitated with 20% (w/v) TCA, washed with acetone, solubilized in loading buffer containing 8 M urea, and then subjected to SDS-PAGE without prior heat treatment. After electrophoresis, the proteins were transferred to a nitrocellulose membrane and subjected to immunoblot analysis using an anti-SptA polyclonal antibody, as described previously (Du et al., 2015).

## Results

### Deletion of *sptA* Affects the Growth Characteristics of *Natrinema* sp. J7-2

**FIGURE 1:**
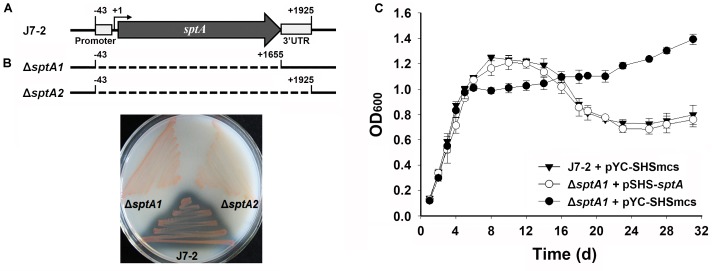
Phenotypic characterization of *Natrinema* sp. J7-2 and its mutants. **(A)** Schematic view of the s*ptA* gene in strain J7-2 and the deletion regions (dashed lines) in mutants Δ*sptA1* and Δ*sptA2*. The gray arrow indicates the *sptA* ORF. The positions of the 5′-end of the core promoter (–43), the transcriptional start site (+1), the 3′-end of the *sptA* ORF (+1655), and the 3′-end of the *sptA* transcript region (+1925) are indicated. The position of the 3′ untranslated region (3′UTR, +1656 to +1925) is also shown. **(B)** Detection of protease production. The strains were grown on 23% MGM agar plates containing 1% skim milk at 37°C for 14 days. **(C)** Growth phenotypes. Strain J7-2 and the Δ*sptA1* mutant harboring the blank vector pYC-SHSmcs or the complementary vector pSHS-*sptA* were grown in 23% MGM with 5 μg ml^-1^ mevinolin at 37°C, and the growth was monitored by the change in OD_600_. Values are expressed as the means and standard deviations (error bars) of three independent experiments.

The core promoter (-43 to -1) and transcript (+1 to +1925) of *sptA* were previously determined (**Figure 1A**) (Tang et al., 2016). In order to investigate the function of SptA, the mutant Δ*sptA1* was created with deletion of the core promoter and entire coding region (-43 to +1655) of *sptA* in *Natrinema* sp. J7-2 (**Figure 1A**). Subsequently, the 3′UTR region of *sptA* in the chromosome of the Δ*sptA1* mutant was further deleted to construct the mutant Δ*sptA2* with deletion of the core promoter and transcript region (-43 to +1925) of *sptA* (**Figure 1A**). The Δ*sptA2* mutant was used as the host to identify *cis*-regulatory elements for *sptA* expression. The purpose of the deletion of the 3′UTR region in Δ*sptA2* is to preclude any possible effect of the 3′UTR region in the chromosome on the expression of *sptA* on the recombinant plasmid that contain the promoter, coding region, and 3′UTR region of *sptA* (see below).

A clear zone was formed around the lawn of parent strain J7-2 but disappeared around that of Δ*sptA1* or Δ*sptA2* grown on the skim milk plate (**Figure 1B**), confirming that SptA is the major extracellular protease of strain J7-2. The parent strain and mutant showed a prominent phenotypic difference in their growth behaviors. When grown in 23% MGM, strain J7-2 harboring a blank vector pYC-SHSmcs (Supplementary Figure S1) (Wang, 2016) showed distinct log, stationary, and death phases (**Figure 1C**). In contrast, the mutant Δ*sptA1* harboring pYC-SHSmcs showed a sharper transition from log phase to stationary phase, and the OD_600_ value did not decrease up to 31 days of cultivation (**Figure 1C**). In a complementation experiment, the mutant Δ*sptA1* harboring the vector pSHS-*sptA* for *sptA* expression showed a growth profile similar to that of strain J7-2 carrying pYC-SHSmcs (**Figure 1C**) and exhibited extracellular proteolytic activity (data not shown). These results suggest that SptA plays an important role in the growth characteristics of *Natrinema* sp. J7-2, particularly in the capacity of the cells to enter the stationary and death phases.

**FIGURE 2:**
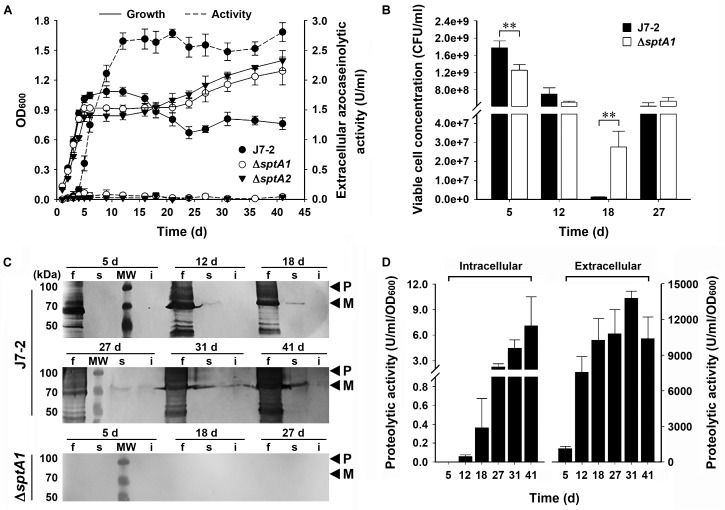
The effect of SptA on the growth and survival of *Natrinema* sp. J7-2 cultivated in 23% MGM at 37°C. **(A)** Growth and extracellular protease production. The growth of strain J7-2 or its *sptA*-deletion mutants (Δ*sptA1* and Δ*sptA2*) was monitored by the change in OD_600_, and the culture supernatants were subjected to azocaseinolytic activity assay. **(B)** Viable cell counting. Aliquots taken from the cultures were appropriately diluted, plated on 18% MGM agar plates, and then incubated at 37°C for 10 days to determine the numbers of viable cells. **(C,D)** Immunoblot analysis and intracellular proteolytic activity assay. At the time points indicated, the culture supernatants (f), cell extracts (s), and cell debris (i) from 500 μl of the cultures were subjected to anti-SptA immunoblot analysis **(C)**. Note that SptA and its derivatives are also absent in the samples of Δ*sptA1* at day 12, 31, and 41 by anti-SptA immunoblot analysis (data not shown). Using suc-AAPF-pNA as the substrate, the proteolytic activities of the culture supernatants and the cell extracts were determined **(D)**. Values are expressed as the means and standard deviations (error bars) of three independent experiments **(A,B,D)** (^∗∗^*P* < 0.01). The positions of the proform (P) and the mature form (M) of SptA on the gels are indicated **(C)**.

We next compared the growth properties of strain J7-2 and its *sptA*-deletion mutants without recombinant plasmids. When grown in 23% MGM, strain J7-2 successively entered the log, stationary, and death phases before 24 days; but after that, the OD_600_ value did not decrease further up to 41 days (**Figure 2A**). In comparison, the Δ*sptA1* and Δ*sptA2* mutants not only showed a sharper transition from log phase to stationary phase but also reached a higher OD_600_ value after 18 days (**Figure 2A**). Consistent with our previous report (Du et al., 2015), extracellular azocaseinolytic activity increased sharply in late log phase and reached its highest level during stationary phase in strain J7-2, whereas it was negligible over the entire growth period in the Δ*sptA1* and Δ*sptA2* mutants (**Figure 2A**). During late log phase, strain J7-2 showed higher OD_600_ values and viable cell numbers than the Δ*sptA1* mutant (e.g., 5 days, **Figures 2A,B**), suggesting that SptA has a positive correlation with the growth of strain J7-2 at this stage. The protein bands with migrations consistent with the proform and mature form of SptA were present in the culture supernatants of strain J7-2, but could not be detected in those of the Δ*sptA1* mutant by immunoblot analysis with the anti-SptA polyclonal antibody (**Figure 2C**). The other bands detected by the anti-SptA polyclonal antibody in the culture supernatants of strain J7-2 may represent the degradation products of the proform and mature form of SptA (**Figure 2C**). The counts of viable cells showed that both strain J7-2 and the Δ*sptA1* mutant entered the death phase at around 12 days (**Figure 2B**). Notably, compared with the Δ*sptA1* mutant, strain J7-2 displayed a sharper decrease in viable-cell number during the death phase (12–18 days, **Figure 2B**). Immunoblot analysis (**Figure 2C**) revealed that mature SptA accumulated within J7-2 cells, but not within Δ*sptA1* cells, as the culture entered the death phase. Meanwhile, proteolytic activity was detected in the intracellular fraction of strain J7-2 (**Figure 2D**), but not in that of the Δ*sptA1* mutant using suc-AAPF-pNA as the substrate (data not shown). These results possibly indicate that the intracellular accumulation of active mature SptA is involved in the transition of strain J7-2 from stationary phase to death phase. In addition, the OD_600_ value of strain J7-2 decreased during the death phase while that of the Δ*sptA1* mutant did not (**Figure 2A**), suggesting a possible contribution of SptA to cell lysis of strain J7-2.

Interestingly, following the death phase, both strain J7-2 and the Δ*sptA1* mutant exhibited a second stage of cell multiplication (18–27 days, **Figure 2B**). During the death phase, the cells of both strains did not form aggregates as evidenced by phase-contrast microscopy (Supplementary Figure S2), suggesting that the observed second stage of cell multiplication is not due to disaggregation of the cells. A reasonable explanation for this phenomenon is that after the death phase, the decrease in the number of viable cells reduces the competition between cells for limiting nutrients, allowing cell multiplication. Meanwhile, the remaining viable cells of both strains could use the nutrients released by the dead cells for growth. Nevertheless, during the second growth stage (18–27 days), the number of viable Δ*sptA1* cells increased only ∼ 20-fold (2.77 × 10^7^ to 5.40 × 10^8^ CFU/ml), while that of viable J7-2 cells increased ∼ 320-fold (1.33 × 10^6^ to 4.24 × 10^8^ CFU/ml; **Figure 2B**). This result suggests that SptA facilitates the viable cells to scavenge the nutrients derived from the dead cells. Both the amount of mature SptA (**Figure 2C**) and the proteolytic activity (**Figure 2D**) in the extracellular fraction were much higher than those in the intracellular fraction, and the extracellular protease activity of strain J7-2 remained at a high level following the death phase (**Figures 2A,D**). In this context, the extracellular SptA plays an important role in degrading proteins of dead cells into peptides or amino acids, which serve as nutrients for living cells.

### The 5′-Flanking Sequence of the *sptA* Core Promoter Contains Positive and Negative *Cis*-Regulatory Regions

**FIGURE 3:**
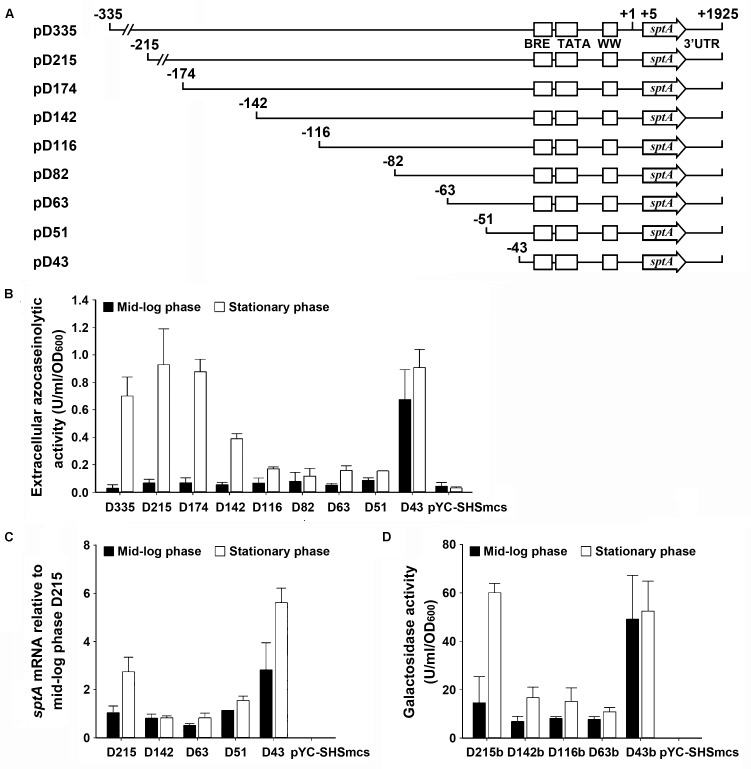
Deletion analysis of the 5′ flanking sequence of *sptA*. **(A)** Schematic view of the deletion constructs. The BRE motif, TATA box, and WW motif are shown as boxes. The positions of the 5′-end position of each construct, the transcriptional start site (+1), the 5′-end of the *sptA* ORF (+5), and the 3′-end of the *sptA* transcript region (+1925) are indicated. The ORF region of *sptA* is presented as an arrow. **(B,C)** Promoter activity assay of the constructs with *sptA* as a reporter gene. The Δ*sptA2* mutants harboring recombinant plasmids (pD335 to pD43) or blank vector (pYC-SHSmcs) were grown in 23% MGM with 5 μg ml^-1^ mevinolin at 37°C. The mid-log and stationary phase culture supernatants were subjected to azocaseinolytic activity assay **(B)**. The levels of *sptA* transcripts of mid-log and stationary phase cells were determined by qRT-PCR analysis using 16S rRNA as an internal control, and the transcript levels of the deletion constructs were determined relative to that of construct D215 at mid-log phase **(C)**. **(D)** Promoter activity assay of the constructs with *bgaH* as a reporter gene. The Δ*sptA2* mutants harboring recombinant plasmids (pD215b to pD43b) or blank vector (pYC-SHSmcs) were grown in 23% MGM with 5 μg ml^-1^ mevinolin at 37°C. The cell extracts of mid-log and stationary phase cultures were subjected to β-galactosidase activity assay. Values are expressed as the means and standard deviations (error bars) of three independent experiments **(B–D)**.

The production of SptA in strain J7-2 was not only growth-phase dependent but also repressed by the presence of ammonium in the culture medium (Supplementary Figure S3), suggesting that *sptA* expression is under strict regulation in this haloarchaeon. To probe possible *cis*-regulatory regions for effects on *sptA* expression, a series of deletion mutants of the 5′-flanking region of the *sptA* promoter were constructed and inserted into the pYC-SHSmcs vector, with *sptA* itself as a reporter gene (**Figure 3A**). The resulting plasmids were introduced into Δ*sptA2* cells to determine the promoter activity based on the extracellular azocaseinolytic activity and the *sptA* transcript level. At the mid-log phase, the cells carrying the D335 construct showed a very low level of background azocaseinolytic activity similar to that of Δ*sptA2* cells carrying a blank pYC-SHSmcs vector. The sequential shortening of the sequence from bp -335 (D335) to bp -51 (D51) did not cause a notable change in the activity (**Figure 3B**); however, the further deletion of eight nucleotides (D43) resulted in a remarkable increase in the azocaseinolytic activity during the mid-log phase (**Figure 3B**). During the stationary phase, cells carrying the D335, D215, and D174 constructs exhibited similar strong azocaseinolytic activities; however, the shortening of the sequence to bp -116 (D116) led to a remarkable decrease in the azocaseinolytic activity (**Figure 3B**). Subsequently, representatives of the constructs with different azocaseinolytic activity levels were selected for verification of promoter activity by measuring their mRNA levels and/or by using the halophilic β-galactosidase-encoding gene *bgaH* as a reporter gene. The mRNA levels of selected constructs were quantified by qRT-PCR, showing that the transcript levels of the constructs (**Figure 3C**) were consistent with the azocaseinolytic activity levels (**Figure 3B**). The β-galactosidase activity levels of the constructs with the *bgaH* reporter (**Figure 3D**) were essentially in agreement with the azocaseinolytic activity levels of the corresponding constructs with the *sptA* reporter (**Figure 3B**). These results suggest that the region from bp -51 to bp -43 contains *cis*-acting element(s) required for negative regulation of *sptA* expression during the mid-log phase, while the region from bp -174 to bp -116 contains *cis*-acting element(s) responsible for positive regulation of *sptA* expression during the stationary phase.

### Multiple Positive and Negative *Cis*-Regulatory Elements Mediate the Growth Phase-Dependent Expression of *sptA*

**FIGURE 4:**
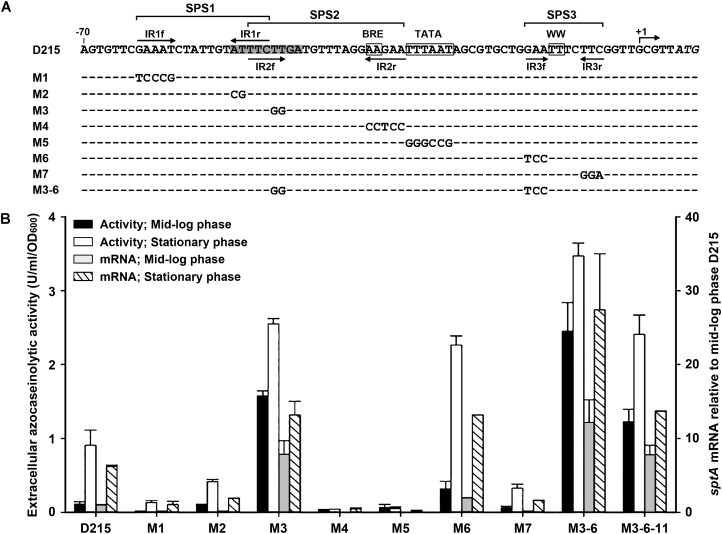
Site-directed mutational analysis of the promoter region of *sptA*. **(A)** Sequences of the promoter regions of the construct D215 and its mutants. The BRE motif, TATA box, and WW motif are boxed. The position of the transcriptional start site (+1) is indicated. The inverted repeat sequences (IR1f/IR1r, IR2f/IR2r, and IR3f/IR3r) are indicated by arrows. The region from bp –51 to bp –43 is shaded. The regions of three semi-palindromic sequences (SPS1, SPS2, and SPS3) are shown. In the sequences of the mutants (M1 to M3-6), the mutated and unaltered nucleotides are shown in letters and dashes, respectively. **(B)** Promoter activity assay. The Δ*sptA2* mutants harboring the recombinant plasmids were grown in 23% MGM with 5 μg ml^-1^ mevinolin at 37°C. The mid-log and stationary phase culture supernatants were subjected to azocaseinolytic activity assay. The levels of *sptA* transcripts of mid-log and stationary phase cells were determined by qRT-PCR analysis using 16S rRNA as an internal control, and the transcript levels of the mutants were determined relative to that of construct D215 at mid-log phase. Values are expressed as the means and standard deviations (error bars) of three independent experiments.

The results of deletion analysis showed that negative *cis*-regulatory element(s) are present upstream of the *sptA* core promoter (**Figure 3**). A sequence analysis revealed two pairs of perfect inverted repeats (IR1f/IR1r and IR2f/IR2r) upstream of the TATA box, as well as a pair of inverted repeats (IR3f/IR3r) flanking the WW motif (**Figure 4A**). These repeats form three semi-palindromic sequences (SPSs): GAAATN_7_ATTTC (SPS1), TTCTTN_10_AAGAA (SPS2), and GAAN_4_TTC (SPS3). Notably, the 5′-half of SPS2 (IR2f) overlaps with the 3′-half of SPS1 (IR1r) by three nucleotides (**Figure 4A**). Considering that the DNA-binding motifs of archaeal transcriptional regulators are almost invariably semi-palindromic in nature (Peeters et al., 2013), we performed a mutational analysis of the SPSs to investigate their possible roles in the regulation of *sptA* expression. For this analysis, we determined the *sptA* promoter activity by measuring the transcript levels and also the extracellular azocaseinolytic activity (**Figure 4B**).

Based on the D215 construct, which was defined as the wild type, the mutation (M3) of the 5′-half of SPS2 (IR2f) without any change to IR1r (**Figure 4A**) led to a remarkable increase in the promoter activity, particularly during the mid-log phase (**Figure 4B**), implying that SPS2 is a negative *cis*-regulatory element. Because the 3′-half of SPS2 (IR2r) covers the BRE motif (**Figure 4A**), it is not astonishing that the mutation (M4) of IR2r caused an almost complete loss of the promoter activity (**Figure 4B**). As expected, the promoter activity was almost abolished by the disruption of the TATA box (M5; **Figure 4B**). In the case of SPS1, the mutation of either the 5′-half (IR1f; M1) or the 3′-half (IR1r; M2) without any change to IR2f (**Figure 4A**) resulted in a remarkable decrease in the promoter activity (**Figure 4B**), suggesting that SPS1 is a positive *cis*-regulatory element. These results demonstrate that the overlapping SPS1 and SPS2 act as positive and negative *cis*-regulatory elements, respectively.

SPS3 is located immediately upstream of the transcriptional start site (**Figure 4A**). The mutation (M6) of the 5′-half of SPS3 (IR3f) led to an increase of the promoter activity (**Figure 4B**), indicating that the semi-palindromic sequence SPS3 represents a negative *cis*-regulatory element. However, the promoter activity of the mutant (M7) with mutations in the 3′-half of SPS3 (IR3r) was decreased rather than increased (**Figure 4B**). One possible explanation for this is that the mutation of IR3r adjacent to the transcriptional start site may affect a putative proximal promoter element or an initiator element that is essential for transcription (Johnsen et al., 2015). When the M6 and M3 mutations were combined with each other, the resulting mutant (M3-6) showed a further increase in the promoter activity (**Figure 4B**), reflecting the cumulative roles of SPS2 and SPS3 in the negative regulation of *sptA* expression.

### A Distant *Cis*-Regulatory Element Acts as an Enhancer to Promote *sptA* Expression

**FIGURE 5:**
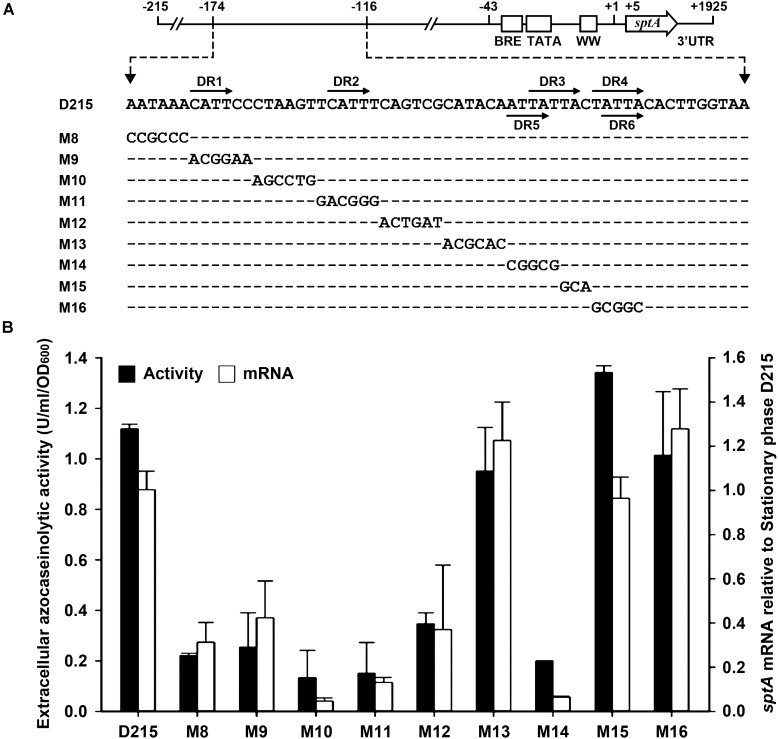
Site-directed mutational analysis of the enhancer region of *sptA*. **(A)** Sequences of the enhancer regions (–174 to –124) of the construct D215 and its mutants. The direct repeat sequences (DR1/DR2, DR3/DR4, and DR5/DR6) are indicated by horizontal arrows. In the sequences of the mutants (M8 to M16), the mutated and unaltered nucleotides are shown in letters and dashes, respectively. **(B)** Promoter activity assay. The Δ*sptA2* mutants harboring the recombinant plasmids were grown in 23% MGM with 5 μg ml^-1^ mevinolin at 37°C. The stationary phase culture supernatants were subjected to azocaseinolytic activity assay. The levels of *sptA* transcripts in stationary phase cells were determined by qRT-PCR analysis using 16S rRNA as an internal control, and the transcript levels of the mutants were determined relative to that of the construct D215. Values are expressed as the means and standard deviations (error bars) of three independent experiments.

According to the results of our deletion analysis (**Figure 3**), the 5′-flanking sequence of *sptA* contains a distant *cis*-regulatory region (–174 to –116) that increases *sptA* expression. A sequence analysis of this region revealed three pairs of direct repeats (DR1/DR2, DR3/DR4, and DR5/DR6; **Figure 5A**), which we analyzed further by scanning mutagenesis. Based on the D215 construct, the mutation of DR1 (M9), DR2 (M11), or the sequences adjacent to DR1 and DR2 (M8, M10, and M12) decreased both the extracellular azocaseinolytic activity and the transcript level by more than 60% in stationary phase (**Figure 5B**). The mutation of the sequence covering DR5 and the first three nucleotides of DR3 (M14) also caused an ∼ 80–90% decrease in both the azocaseinolytic activity and the transcript level, whereas the mutation of the last two nucleotides of DR3 (M15) and the sequence covering DR4 and DR6 (M16) did not affect the promoter activity (**Figure 5B**). During mid-log phase, all of the mutants exhibited low levels of extracellular azocaseinolytic activity similar to that of the D215 construct (data not shown). Those results suggest that the region from bp -174 to bp -134 containing the direct repeats DR1 and DR2 is a positive *cis*-regulatory element (named UAS1) for *sptA* expression.

We next investigated whether the regulatory role of UAS1 in *sptA* expression is growth-phase dependent. We introduced the M11 mutations into the M3-6 mutant, which exhibited an increase in promoter activity at both the mid-log and stationary phases (**Figure 4**). The resulting mutant (M3-6-11) showed a decrease in promoter activity at both the mid-log and stationary phases in comparison with the M3-6 mutant (**Figure 5B**). Therefore, UAS1 acts as a distant enhancer to promote *sptA* expression, and its action is independent of the growth phase.

## Discussion

Halolysin SptA is the major extracellular protease of *Natrinema* sp. J7-2, and its production is growth-phase dependent. The growth phase-dependent production of halolysin has been described in some haloarchaea such as *Ncc. occultus* (Studdert et al., 1997), *Nab. asiatica* (Kamekura and Seno, 1990), and *Nab. magadii* (Paggi et al., 2010). It has been proposed that the extracellular accumulation of the halolysin Nep at elevated cell densities allows more efficient scavenging of protein substrates in the natural environment of *Nab. magadii* (Paggi et al., 2010). We found that SptA functions both extracellularly and intracellularly and exerts different influences on *Natrinema* sp. J7-2 during different growth phases. Strain J7-2 starts to produce and secrete SptA during late log phase when easily available nutrients become depleted. The extracellular SptA-mediated degradation of protein substrates facilitates the growth of strain J7-2 by enabling the nutrients in the medium to be utilized more efficiently. This is supported by the findings that the *sptA*-deletion mutant showed a sharper transition from log phase to stationary phase and a lower growth rate during late log phase compared with strain J7-2. The extracellular production of SptA in strain J7-2 peaks when the culture enters the stationary phase. Meanwhile, small amounts of SptA gradually accumulate within J7-2 cells as the culture entered the death phase, most likely because of functional deterioration of the protein transport system in aging cells. Consistent with our previous report (Du et al., 2015), proteolytic activity assays and immunoblot analysis showed that intracellular SptA is capable of autocatalytic activation. It is noteworthy that strain J7-2 displayed a sharper transition from stationary phase to death phase in comparison with the *sptA*-deletion mutant. This is apparently due to the intracellular activation of SptA and associated proteolytic damage to cellular proteins. In addition, the OD_600_ value of the *sptA*-deletion mutant was higher than that of strain J7-2 during and after the death phase (18–41 days, **Figure 2A**), suggesting that SptA is involved in cell lysis in strain J7-2. Therefore, the extracellular production of SptA during the late log phase promotes the growth of strain J7-2, while the intracellular accumulation of SptA during stationary phase helps the haloarchaeon to enter the death phase. The promotion of cell death and lysis by SptA during the death phase increases the amount of dead cell-derived nutrients that are available for surviving cells. In addition, the SptA-mediated degradation of dead cell-derived proteins enables the surviving cells to scavenge those protein substrates more efficiently.

The growth phase-dependent production of SptA relies on strict regulation of *sptA* expression. The addition of ammonium to the culture medium repressed SptA production, suggesting that SptA production may be regulated by a nitrogen catabolite repression mechanism, as has been proposed for the halolysin Nep of *Nab. magadii* (D’Alessandro et al., 2007). Although the regulatory proteins involved in the expression of halolysins remain to be determined, we have identified multiple positive and negative *cis*-regulatory elements that mediate the growth phase-dependent production of SptA. Mutational analyses revealed that SPS2 and SPS3, which cover the BRE and WW motifs, respectively, are responsible for the negative regulation of *sptA* transcription in *Natrinema* sp. J7-2. Similarly, a negative regulatory element with a semi-palindromic nature resides immediately upstream of the BRE motif in the *phaRP* promoter in *Hfx. mediterranei* (Cai et al., 2015), and a negative regulatory SPS covering the WW motif has been identified in the *xacR* promoter in *Hfx. volcanii* (Johnsen et al., 2015). Two major repression mechanisms have been described in archaea (Peeters et al., 2013). In the first, the binding of a repressor at a site overlapping the BRE and TATA box impairs promoter access for TFB and TBP through steric hindrance. In the second, a repressor binds to a site downstream of the TATA box to affect the recruitment of RNAP. In this context, SPS2 and SPS3 most likely act as two binding sites of repressors and function cumulatively to negatively regulate *sptA* expression in *Natrinema* sp. J7-2. The mutation of either SPS2 (M3) or SPS3 (M6) leads to an increase in *sptA* expression during mid-log phase, suggesting that both SPSs are responsible for the repression of *sptA* transcription before the J7-2 culture enters the late log phase. Our mutational analysis also revealed a positive *cis*-acting element, SPS1, which may represent a binding site of an activator. In archaea, activators generally interact with TFB and/or TBP to promote the assembly of the pre-initiation complex (PIC) that is necessary for gene transcription (Bleiholder et al., 2012; Peeters et al., 2013). Interestingly, the positive regulatory SPS1 partially overlaps with the negative regulatory SPS2. It is very likely that the binding of the activator to SPS1 can prevent the binding of the repressor to SPS2 via steric hindrance and then promote PIC assembly. Besides SPS1, we identified another positive *cis*-regulatory element, UAS1, which is localized ∼100 bp upstream of the *sptA* core promoter and acts as a distant enhancer. In bacteria, enhancer-binding proteins (EBPs) typically bind at enhancer sites 80 bp to 150 bp upstream of the promoter; the formation of an enhancer-promoter loop allows the EBPs to interact with the RNAP-σ^54^ holoenzyme and thus activate the promoter, which is known as the looping mechanism (Liu et al., 2001; Bush and Dixon, 2012; Kulaeva et al., 2012). Accordingly, UAS1 appears to act as a distant enhancer of *sptA* transcription and functions in a manner similar to that of bacterial enhancers. Notably, the mutation of UAS1 in the M3-6 mutant (M3-6-11) reduced *sptA* expression during both the mid-log and the stationary phases, clearly indicating that the regulatory role of UAS1 is not growth-phase dependent. It is plausible that the enhancer UAS1 will exert its function upon the formation of the PIC.

**FIGURE 6:**
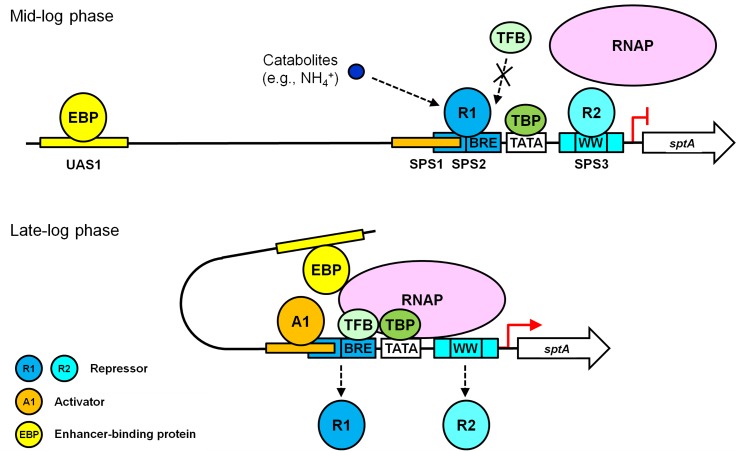
A working model of the roles of the multiple *cis*-acting elements in the regulation of *sptA* expression in *Natrinema* sp. J7-2.

Based on our results and known principles of transcriptional repression and activation (Bush and Dixon, 2012; Kulaeva et al., 2012; Peeters et al., 2013), we propose a working model for the roles of the multiple *cis*-acting elements in the regulation of growth phase-dependent SptA production in *Natrinema* sp. J7-2 (**Figure 6**). During the mid-log phase, the presence of nitrogen catabolites (e.g., NH_4_^+^) promotes repressors binding to SPS2 and SPS3. The binding of a repressor (R1) to SPS2 prevents TFB from approaching the BRE motif through steric hindrance, while the occupation of SPS3 by another repressor (R2) affects the recruitment of RNAP. Under this circumstance, the PIC cannot be assembled properly, and *sptA* transcription is blocked. When the J7-2 culture enters the late log phase, an activator (A1) competes with the repressor R1 for the overlapping region between SPS1 and SPS2. The binding of the activator A1 to SPS1 leads to the release of R1 from SPS2, allowing TFB to bind the BRE motif. The SPS1-bound A1 may also play a role in recruiting TFB/TBP in a manner similar to that of other archaeal activators (Peng et al., 2011). During the recruitment of RNAP by TFB/TBP, RNAP may compete with the repressor R2 for interaction with the WW motif, and the release of R2 from SPS3 facilitates the assembly of the PIC. Subsequently, the UAS1-bound EBP interacts with the PIC through the looping mechanism to promote *sptA* transcription. Although our working model explains the roles of the *cis*-regulatory elements of *sptA*, further study is warranted to identify and characterize the regulatory proteins in order to elucidate the mechanism of the growth phase-dependent production of SptA.

In summary, our results demonstrate that halolysin SptA, the major extracellular protease involved in the degradation of protein substrates in the natural environment of *Natrinema* sp. J7-2, also participates in cell death and lysis to affect the growth characteristics of the haloarchaeon. Moreover, we found that multiple positive and negative *cis*-regulatory elements collaboratively mediate the growth phase-dependent expression of *sptA*, thereby enabling the enzyme to exert its function properly during the different growth stages of *Natrinema* sp. J7-2. This study provides new insight into the physiological role of halolysins and the mechanism by which halolysin transcription is regulated in haloarchaea.

## Author Contributions

ML, JY, SM, and XW conducted the experiments. ML, X-FT, and BT analyzed and interpreted the results and contributed to writing the paper.

## Conflict of Interest Statement

The authors declare that the research was conducted in the absence of any commercial or financial relationships that could be construed as a potential conflict of interest.

## Footnotes

1 http://www.haloarchaea.com/resources/halohandbook/index.html
