# Supplementary material for: Halolysin SptA, a Serine Protease, Contributes to Growth-Phase Transition of Haloarchaeon Natrinema sp. J7-2, and Its Expression Involves Cooperative Action of Multiple Cis-Regulatory Elements
Source: Front Microbiol. 2018 Aug 3;9:1799. doi: 10.3389/fmicb.2018.01799 (PMC6085418; doi:10.3389/fmicb.2018.01799)
Supplement: Supplementary file 1 [file Presentation_1.pdf]

SUPPLEMENTAL MATERIAL

Halolysin SptA, a serine protease, contributes to growth-phase transition of haloarchaeon *Natrinema* sp. J7-2, and its expression involves cooperative action of multiple *cis*-regulatory elements

Moran Li,[a] Jing Yin,[a] Sha Mei,[a] Xuhong Wang,[a] Xiao-Feng Tang,[a,b]# Bing Tang,[a,b]#

State Key Laboratory of Virology, College of Life Sciences, Wuhan University, Wuhan, China[a]; Hubei Provincial Cooperative Innovation Center of Industrial Fermentation, Wuhan, China[b]

Running title: Function and regulation of halolysin SptA

#Address correspondence to Xiao-Feng Tang, tangxf@whu.edu.cn; Bing Tang, tangb@whu.edu.cn.

TABLE S1. Strains and plasmids used in this study

Strain or plasmid	Description	Reference or source
**Strains**		
E. coli		
DH5α	F^- φ80d/lacZΔM15 Δ(*lacZYA-argF*) U169 *deoR recA*1 *hsdR*17(rk^- mk^-) *phoA supE*44 λ^- *thi*-1 *gyrA*96 *relA*1	Novagen
JM110	*dam dcm supE*44 *hsdR*17 *thi leu rpsL*1 *lacY galK galT ara tonA thr tsx* (*lac-proAB*)/F' [*traD*36 $proAB^+$ $lacI^q$ *lacZ*ΔM15]	Novagen
Natrinema sp.		
J7-2	A subculture of *Natrinema* sp. J7 (CCTCC AB91141) lacking the plasmid pHH205	(1)
Δ*sptA1*	J7-2 mutant with a deletion of the core promoter and ORF region (-43 to +1655) of *sptA*	This study
Δ*sptA2*	J7-2 mutant with a deletion of the core promoter and transcript region (-43 to +1925) of *sptA*	This study
**Plasmids**		
pNBK07	Haloarchaeal nonreplicative plasmid, Mev^R, Amp^R	(2)
pNBK-*Aud*	Knockout plasmid for the core promoter and ORF region of *sptA*	This study
pNBK-3'UTR*ud*	Knockout plasmid for the 3’UTR region of *sptA*	This study
pYC-SHSmcs	*E. coli-Natrinema* sp. J7-2 shuttle vector, Mev^R, Amp^R	(2)
pSHS-*sptA*	pYC-SHSmcs-derived vector containing the core promoter and ORF region of *sptA* (-43 to +1655)	This study
pD355	pYC-SHSmcs-derived vector containing *sptA* transcript and its 5’-flanking region (-335 to +1925)	This study
pD215	pYC-SHSmcs-derived vector containing *sptA* transcript and its 5’-flanking region (-215 to +1925)	This study
pD174	pYC-SHSmcs-derived vector containing *sptA* transcript and its 5’-flanking region (-174 to +1925)	This study

pD142	pYC-SHSmcs-derived vector containing *sptA* transcript and its 5'-flanking region (-142 to +1925)	This study
pD116	pYC-SHSmcs-derived vector containing *sptA* transcript and its 5'-flanking region (-116 to +1925)	This study
pD82	pYC-SHSmcs-derived vector containing *sptA* transcript and its 5'-flanking region (-82 to +1925)	This study
pD63	pYC-SHSmcs-derived vector containing *sptA* transcript and its 5'-flanking region (-63 to +1925)	This study
pD51	pYC-SHSmcs-derived vector containing *sptA* transcript and its 5'-flanking region (-51 to +1925)	This study
pD43	pYC-SHSmcs-derived vector containing *sptA* transcript and its 5'-flanking region (-43 to +1925)	This study
pM1 to pM3-6-11	pD215-derived vectors with mutations introduced into 5'-flanking region of *sptA*	This study
pST1	A vector containing haloarchaeal *bgaH* gene, Amp^R, Nov^R	(3)
pD215b	pYC-SHSmcs-derived vector containing *bgaH* ORF and 5'-flanking region (-215 to -1) of *sptA*	This study
pD142b	pYC-SHSmcs-derived vector containing *bgaH* ORF and 5'-flanking region (-142 to -1) of *sptA*	This study
pD116b	pYC-SHSmcs-derived vector containing *bgaH* ORF and 5'-flanking region (-116 to -1) of *sptA*	This study
pD63b	pYC-SHSmcs-derived vector containing *bgaH* ORF and 5'-flanking region (-63 to -1) of *sptA*	This study
pD43b	pYC-SHSmcs-derived vector containing *bgaH* ORF and 5'-flanking region (-43 to -1) of *sptA*	This study

References

1. Feng J, Liu B, Zhang Z, Ren Y, Li Y, Gan F, Huang Y, Chen X, Shen P, Wang L, Tang B, Tang XF. 2012. The complete genome sequence of *Natrinema* sp. J7-2, a haloarchaeon capable of growth on synthetic media without amino acid supplements. PLoS One 7:e41621.
2. Wang Y, Sima L, Lv J, Huang S, Liu Y, Wang J, Krupovic M, Chen X. 2016. Identification, characterization, and application of the replicon region of the halophilic temperate sphaerolipovirus SNJ1. J Bacteriol 198:1952-1964.
3. Tang W, Wu Y, Li M, Wang J, Mei S, Tang B, Tang XF. 2016. Alternative translation initiation of a haloarchaeal serine protease transcript containing two in-frame start codons. J Bacteriol 198:1892-1901.

TABLE S2. Primers used in this study

Primer	Sequence (5'→3') [a]
**Gene knockout**	
SptA-F	ATGTTTAGGAAGAATTTAATAG
SptA-R	CTATCGACCGCGTTCGTCG
SptAus-F	GA*AGATCT*TCTCAACGAACAACCCGACTG
SptAus-R	GCGGCCGCCGCTCGATCAAGAAATACAATAGAT
SptAds-F	ATCTATTGTATTTCTTGATCGAGCGGCGGCCGC
SptAds-R	CCC*AAGCTT*GGGGGAACGACAGTCAGCAAGT
3'UTRus-R	GTGAACTCAGCGGTTTACAAGAAATACAATAGAT
3'UTRds-F	ATCTATTGTATTTCTTGTAAACCGCTGAGTTCAC
3'UTRds-R	CCC*AAGCTT*GGGGGCACGGGCCTCACTGGCCAC
3'UTR-F	CGAACGCGGTCGATAGATGAGCAGCGGAGGCC
3'UTR-R	GGCCTCCGCTGCTCATCTATCGACCGCGTTCG
**Gene complementary expression**	
SptA-BamHI-F	CG*GGATCC*CGATGTTTAGGAAGAATTTAATAGCGTGCTGG
SptA-HindIII-R	CCC*AAGCTT*GGGCTATCGACCGCGTTCGTCGATAGTCATGG
**Truncation of 5'-flanking region of *sptA***	
up335-F	CG*GGATCC*CGACGCGGTCAAACATGGTC
up215-F	CG*GGATCC*CGGATGGTCCGAATCACCCC
up174-F	CG*GGATCC*CGAATAAACATTCCCTAAGTTCATTTC
up142-F	CG*GGATCC*CGTACAATTATTACTATTAC
up116-F	CG*GGATCC*CGACCCTCATCATGGGTACTCG
up82-F	CG*GGATCC*CGGATTATAATATTAGTGTTCG
up63-F	CG*GGATCC*CGGAAATCTATTGTATTTCTTG
up51-F	CG*GGATCC*CGATTTCTTGATGTTTAGGAAG
up43-F	CG*GGATCC*CGATGTTTAGGAAGAATTTAATAGCGTGCTGG
3'UTR-HindIII-R	CCC*AAGCTT*GGGATATAAGGTGTGGATATG
**Replacement of *sptA* with *bgaH***	
up-bgaH-R	TAGCAGACACCAACTGTCATAACGCAACCGAAGAAATTCC
bgaH-HindIII-R	CCC*AAGCTT*GGGTCACTCGGACGCGAGTCC

**Mutation of 5'-flanking region of *sptA***	
m1-R	AAATACAATAG**CGGGA**GAACACTAATA
m2-R	CCTAAACATCAAGAA**CG**ACAATAGATTTCGAACA
m3-R	ATTCTTCCTAAACATC**CC**GAAATACAATAG
m4-R	ACGCTATTAAA**GGAGG**CCTAAACATCA
m5-R	TCCAGCACGCT**CGCCC**TTCTTCCTAAA
m6-R	AACCGAAGAAA**GGA**CAGCACGCTAT
m7-R	ATAACGCAACC**TCC**GAAATTCCAGC
m8-R	CTTAGGGAATG**GGGCGG**CCACAACTCTT
m9-R	AATGAACTTAG**TTCCGT**TTTATTCCACA
m10-R	GACTGAAATGA**CAGGCT**GGAATGTTTAT
m11-R	GTATGCGACTG**CCCGTC**ACTTAGGGAAT
m12-R	ATAATTGTATG**ATCATG**AAATGAACTTA
m13-R	GTAATAGTAATAAT**GTGCGT**CGACTGAAATG
m14-R	GTGTAATAGTA**CGCCG**TGTATGCGACT
m15-R	ACCAAGTGTAATA**TGC**ATAATTGTATGCGA
m16-R	GTTACCAAGTG**GCCGC**GTAATAATTGT
**qRT-PCR**	
SptAQRT-F	CGAACATTGTCCACACGAAC
SptAQRT-R	GGTCACGTTGTCTTCGGAGT
16SrRNAQRT-F	GGTACGGGTTGTGAGAGCAA
16SrRNAQRT-R	CAGTGTAAAGGTTTCGCGCC

[a] Italicized sections indicate restriction enzyme sites. The mutated nucleotides are in bold.

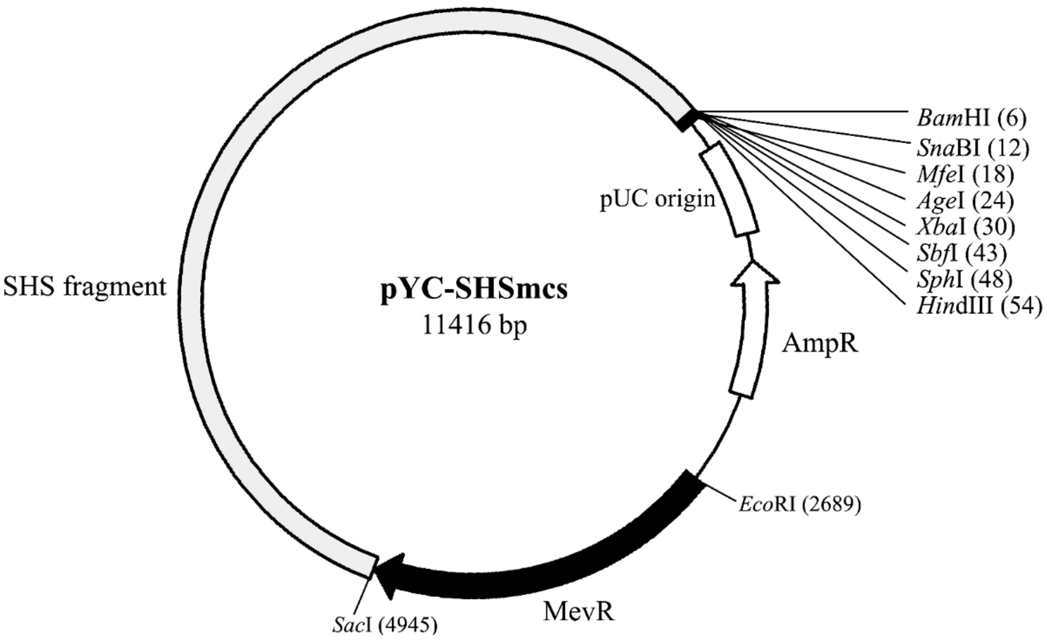

FIG S1 A restriction map of the plasmid pYC-SHSmcs. SHS fragment represents the minimal replicon of SNJ1, which is a temperate haloarchaeal virus identified in *Natrinema* sp. J7.

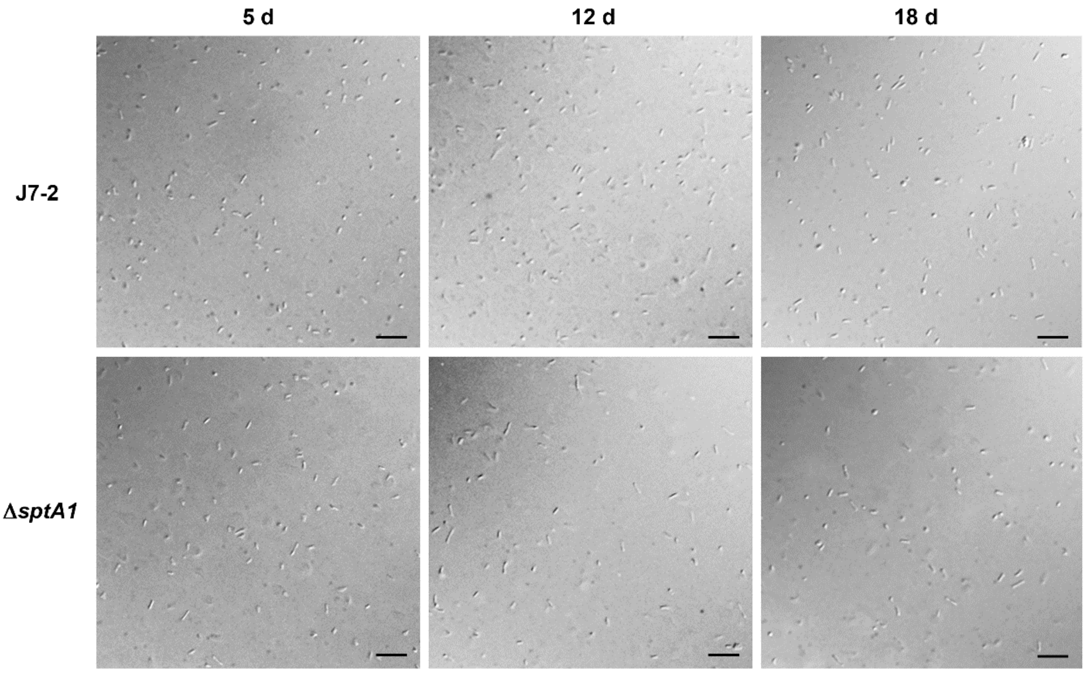

FIG S2 Phase-contrast microscopy of strain J7-2 and mutant Δ*sptA1* grown in 23% MGM. The late-log (5 d) and death (12 d and 18 d) phase cultures were examined. Bars = 10 μm.

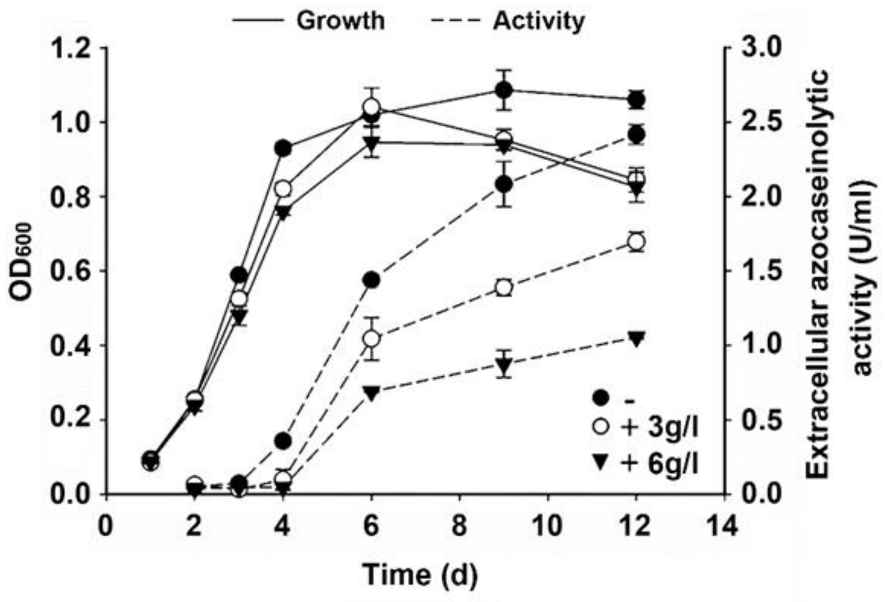

FIG S3 Effects of ammonium on SptA production in *Natrinema* sp. J7-2. Strain J7-2 was cultivated at 37°C in 23% MGM without (-) or with (+) 3 or 6 g/l of NH_4Cl. Haloarchaeal growth was monitored by change in OD_{600}, and the culture supernatants were subjected to azocaseinolytic activity assay. Values are expressed as the means and standard deviations (error bars) of three independent experiments.
